# Hemorrhoids; Prolapsed Rectum; New Operation*Read before the Surgical Section of the Mississippi Valley Medical Association, Detroit, September 3-7, 1895.

**Published:** 1895-10

**Authors:** Merrill Ricketts

**Affiliations:** Cincinnati


### HEMORRHOIDS; PROLAPSED RECTUM; NEW OPERATION.*

BY MERRILL RICKETTS, M. D., CINCINNATI.

The object of this paper is to present means more simple than have heretofore offered for the radical cure of a prolapsed rectum, and the obliteration of hemorrhoids.

It is first necessary to understand the anatomy of the pelvis, and especially the rectum and its immediate environments, and the causes of these two conditions.

It being taken for granted that the practitioner possesses this knowledge, a brief explanation is all that is necessary to understand how to proceed in the first.

HEMORRHOIDS.—After widely dilating the sphincter with the fingers, under surgical anæsthesia, a large semicircular needle is passed subcutaneously from the muco-cutaneous line to the upper border of the pile-bearing area, and then returned to make its exit at the point of entrance; the needle is then removed, and the silk ligature made taut about the venus plexus, and the ends left hanging out. These ligatures may be from one-half to an inch apart, as the case may require. It is not necessary to tie all the varices in this way, as the atrophic changes will necessarily obliterate the remaining ones. No tissue is sacrificed; the mucous membrane remains intact; there is no hemorrhage, no infection, no pain of consequence, and the loss of time practically *nil.*

While the operation is absolutely radical, and without any serious consequences, the sutures are allowed to come away of their own accord.

The operations made in this way for this condition have resulted in an absolute cure, and the patients have experienced but little pain during the life of the ligature.

Prolapsed rectum is treated in the same manner, except a greater number of ligatures are required. If they are properly and evenly adjusted, the atrophy of the tissue is symmetrical, and the pathological condition is relieved without stenosis.

---

* Read before the Surgical Section of the Mississippi Valley Medical Association, Detroit, September 3-7, 1895.

It is, of course, necessary to pass the needle somewhat deeper in cases of prolapsus, than it is in hemorrhoids pure and simple.

The time required, however, for prolapsus is greater than that for hemorrhoids—it sometimes being necessary for a second sitting, at an interval of from four to eight weeks to correct the deformity.

It may be that kangaroo tendons, chromatized catgut, silkworm gut, or silver, will answer the purpose better than silk.

Nothing but experience, however, will settle this question.

Two operations for congenital prolapsus of the rectum to an exaggerated degree have resulted most satisfactorily.

It is to be hoped that this, or some other procedure, will supplant the operation of Whitehead or the so-called infamous American operation. The sooner these two operations are relegated, the better it will be for the laity, and the subsequent operations will be fewer to remunerate the rectal surgeon.

---

PHYTOLACCA DECANDRA FOR EPITHELIOMA.—The method of using the remedy is to bruise the green leaves to a pulpy mass; collect the expressed juice in a shallow receptacle, as a plate; allow it to evaporate to a thick, pasty consistency; spread a portion of this on a piece of silk or other suitable cloth, and apply to the morbid growth.

The plaster should be removed; the part washed twice daily. The remedy causes severe pain. It has a selective action for the morbid tissue; follows out all the irregularities of the epithelioma; causes, as it were, its liquefaction and removal; and then acts as a cicatrizant for the open sore.

As soon as all the morbid tissue is destroyed, a bed of cicatricial tissue begins to spread from the periphery, and as this occurs, the plaster should be cut smaller each day, so as to conform to the size and shape of the surface to be covered by it.

Under this treatment, the writer has seen large epitheliomatous masses destroyed in a few weeks, and nothing but a faint scar left at the place occupied by the growth. In no case was there a recurrence at the original site.

Unlike other remedies, it can be used fearlessly, does not endanger the patient, combines within itself a caustic action and healing property, and requires to be used in the same manner, from beginning to end.—S. C. GOODMAN, in *Medical Journal*.

